# Vascular Complications of Splenectomy in a Patient with Gastric Dieulafoy-like Lesions in Left-sided Portal Hypertension Secondary to Splenic Vein and Artery Thrombosis

**DOI:** 10.7759/cureus.6727

**Published:** 2020-01-21

**Authors:** Mehdi Faraji, Taylor S Harmon, Arya N Bagherpour, Lynsey M Maciolek

**Affiliations:** 1 Radiology, Louisiana State University Health Sciences Center, Shreveport, USA; 2 Radiology, University of Florida College of Medicine, Jacksonville, USA; 3 Radiology, University of Texas Medical Branch, Galveston, USA

**Keywords:** splenic, artery, vein, dieulafoy, splenectomy, embolization

## Abstract

Due to lower clinical significance, the management of Dieulafoy and Dieulafoy-like lesions is less commonly reported than the management of their impending venous equivalent, variceal bleeding. Though Dieulafoy and Dieulafoy-like lesions are often benign, they can become life-threatening in certain clinical scenarios, especially with substantial changes in hemodynamic blood flow, which results in hemorrhage. Post-procedural hemodynamic blood flow should be carefully monitored in patients who receive procedures that drastically alter hemodynamic flow pressures. Factoring in the presence of Dieulafoy and Dieulafoy-like lesions might deepen the complexity of an intuitive surgical or interventional procedure for an experienced operator, and should, therefore, involve the cooperative effort between surgical, interventional, and diagnostic services to appropriately manage the patients. The case we present demonstrates the dire consequences of a routine splenectomy when a considerable change in hemodynamic pressure across benign Dieulafoy-like lesions occurs in a patient with both splenic artery and venous thrombosis.

## Introduction

Dieulafoy lesions account for only 6% of upper gastrointestinal (GI) nonvariceal bleeding and a mere 1-2% of all GI hemorrhages [1]. Although these statistics seem negligible, they cannot be ignored, as there is potential for death secondary to exsanguination from these sites. Overall, it is still important to note that the death rate is relatively low in both GI bleeding and, more specifically, in Dieulafoy lesions, which makes the patient discussed in our study even more of an anomaly [1]. By definition, a Dieulafoy lesion is comprised of dilated submucosal arteries that occupy an area 1-3 mm in radius with various distances from the gastric mucosa. They become life-threatening when there is an erosion of the gastric mucosa and arterial walls, which could lead to massive hemorrhage [1,2]. The cause of the erosion is generally difficult to identify; in many cases, it is secondary to both gastric mucosa atrophy and ischemia due to increased pressure from the distended submucosal arteries [1]. This increased pressure can precipitate reduction in blood flow in the overlying mucosa, inducing ischemia [3]. Pathologically, the mucosa surrounding Dieulafoy lesions is normal, without any aneurysmal, arteriosclerotic, or vasculitis changes. There are also no signs of inflammatory cell infiltration in these regions in general [3].

Current literature seems to endorse a relationship between the ingestion of certain products and the formation of Dieulafoy lesions. These include nonsteroidal anti-inflammatory drugs (NSAIDs), anticoagulants, antiplatelets, and chronic alcohol use. Beyond this, it is reported that diseases that transform the process of angiogenesis, such as cardiac disorders, hypertension, and renal failure, can often manifest these dilated and tortuous submucosal arteries. Dieulafoy lesions can also be found congenitally; so the etiology is not confined to acquired as previously believed. The exact pathogenesis has not been identified yet, but it has been determined that 80 to 95% of Dieulafoy lesions occur within 6 cm of the gastroesophageal junction [1]. This information can aid physicians in finding Dieulafoy lesions, which are difficult to locate when they are nonbleeding or concealed within the troughs of gastric folds. Even though these lesions may lead to a fatal hemorrhagic episode, the mucosal defect is small and without evidence of an ulcer, allowing them to be easily covered by blood or clots. Beyond this, they are often difficult to identify following a bleeding episode due to their tendency to constrict and retract following these hemorrhagic events [4]. We present the case of a patient with both splenic vein thrombosis and splenic artery thrombosis resulting in gastric Dieulafoy-like lesions. Previous studies of patients with left-sided portal hypertension have revealed a predilection for gastric varices with the existence of gastric fundus collaterals and hypertension in the left gastric vein [5]. To our knowledge, there is no existing study involving a patient with both splenic artery and vein thrombosis with Dieulafoy-like lesions and vascular complications post-splenectomy.

## Case presentation

**Figure 1:**
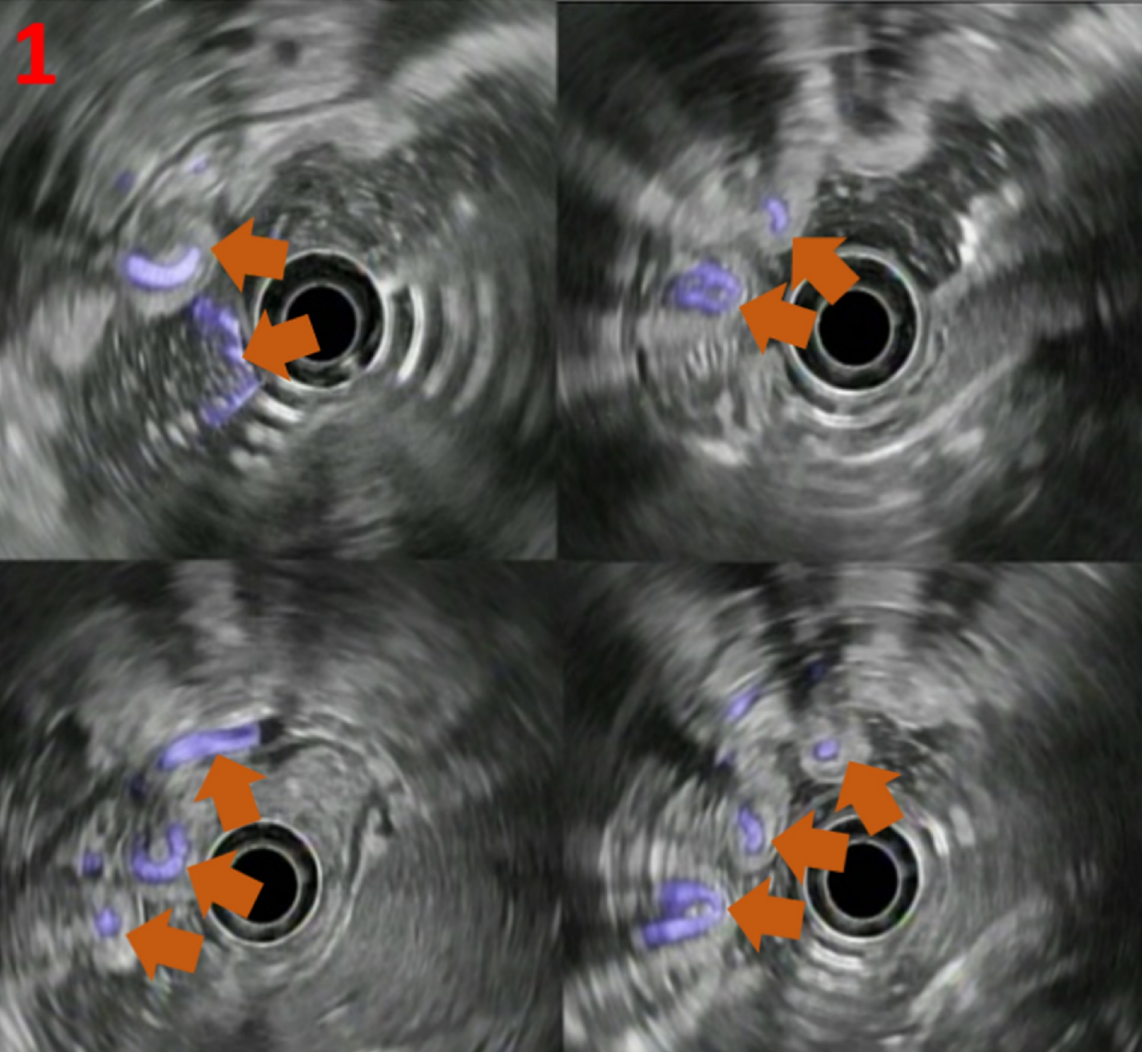
Esophageal ultrasound of varices Esophageal ultrasound with color Doppler signal shows varices (orange arrows) as demonstrated by serpiginous veins (purple fill)

This case looks at a 58-year-old male who was also hepatitis C virus-positive (previously treated in 2005) without cirrhosis. He presented to the emergency room with hematemesis and melena for two days. He underwent urgent esophagogastroduodenoscopy (EGD) and endoscopic ultrasound (EUS) examinations, which showed tubal, anechoic structures in the fundus of the stomach, most likely representing gastric fundal varices (Figure 1).

**Figure 2:**
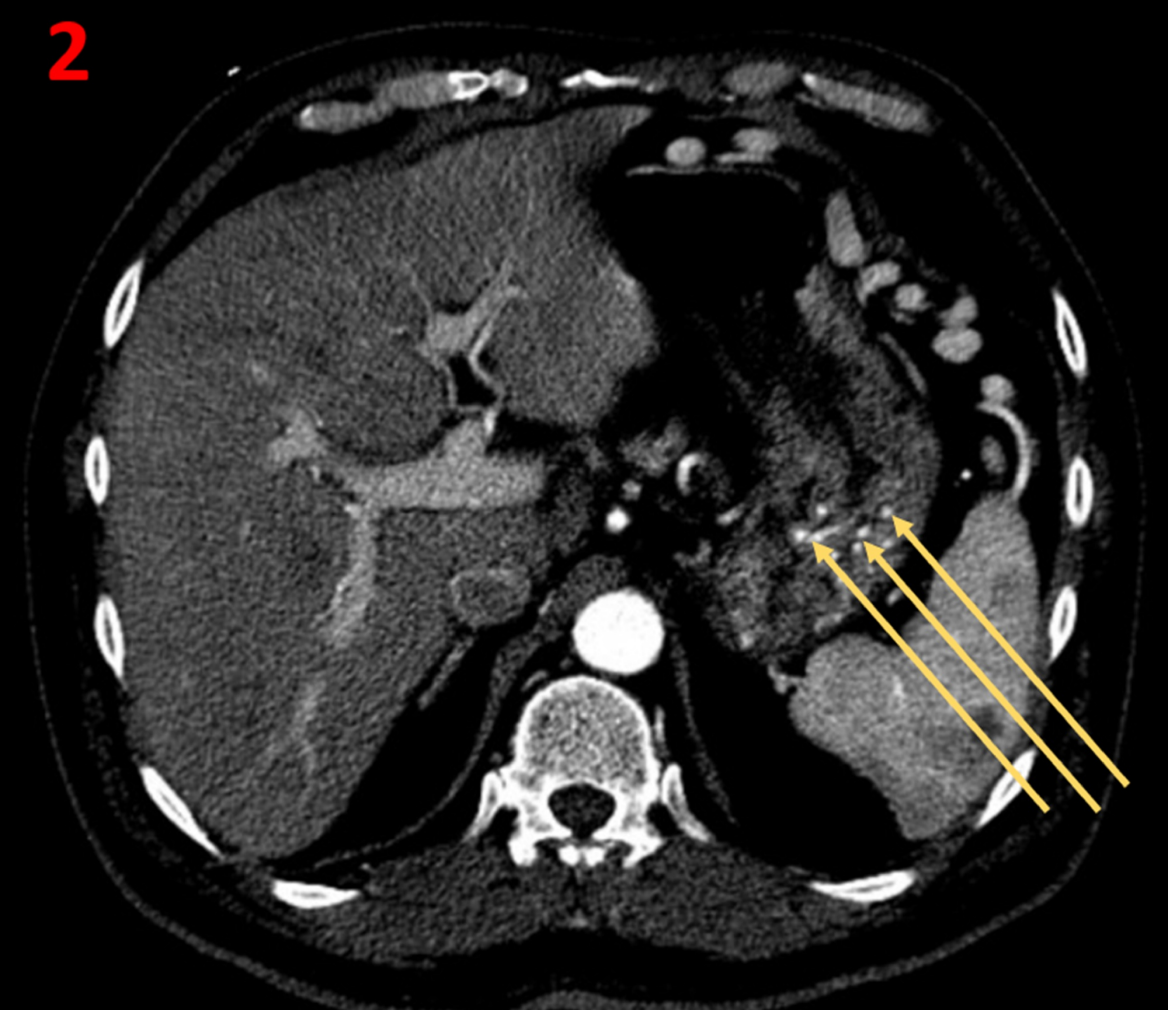
Gastric fundal wall as seen on axial abdominal CT CT: computed tomography Contrast-enhanced axial CT in the arterial phase demonstrates multiple large arteries (yellow arrows) in the gastric fundal wall with some vessels coursing in close approximation to the submucosa

**Figure 3:**
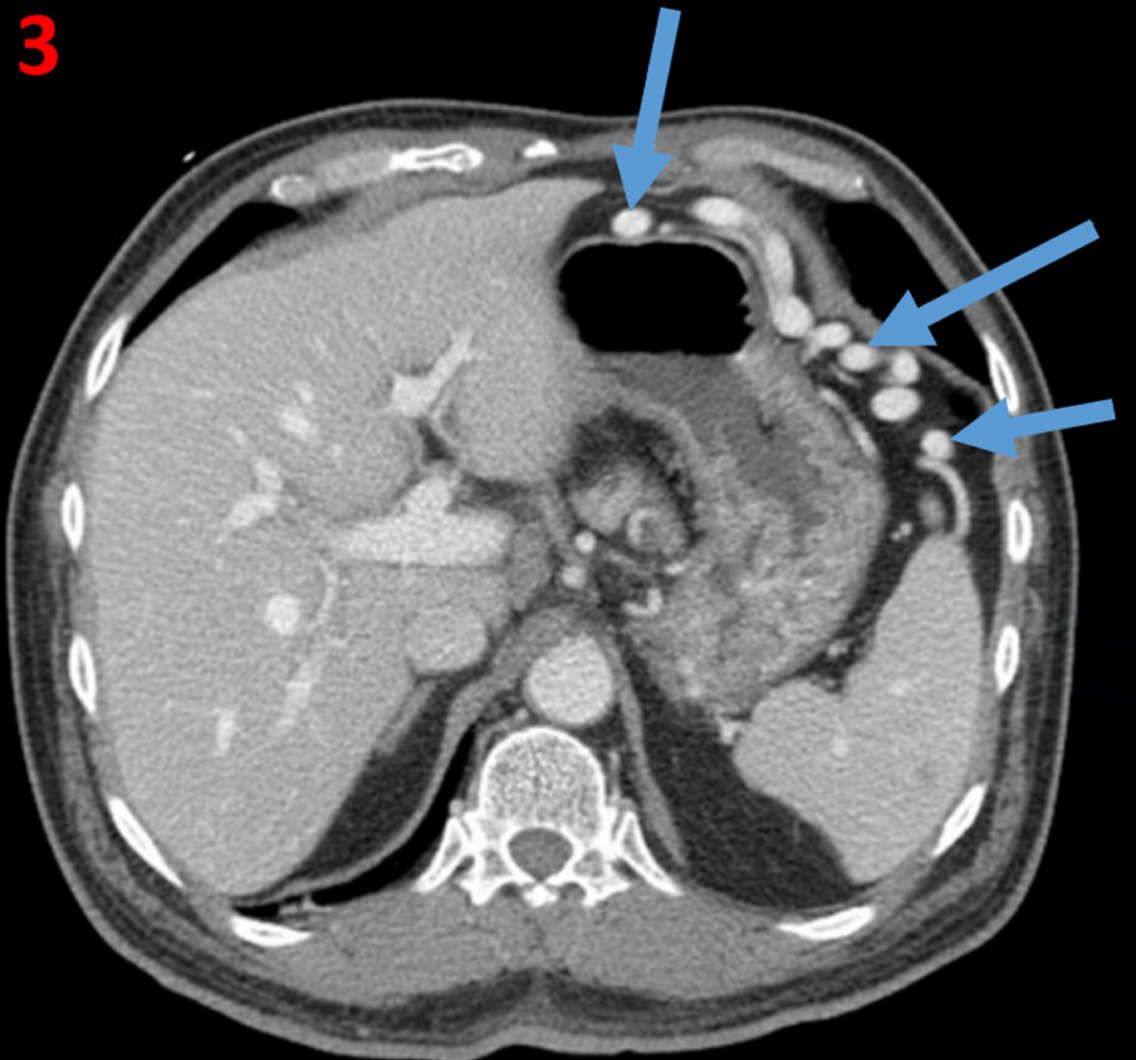
Varix on axial abdominal CT CT: Computed tomography Axial CT demonstrates a large collateral varix (blue arrows) which courses through abdominal mesentery. This varix is providing venous drainage of the spleen due to chronic splenic vein thrombosis

A CT scan of the abdomen and pelvis performed the following day showed a chronically thrombosed splenic vein and artery, with a viable spleen. Arterial supply of the spleen was almost exclusively supplied by the left gastric artery, which resulted in markedly hypertrophied collateral arteries that traversed through the stomach wall (Figure 2). These hypertrophied collaterals arteries were determined to be Dieulafoy-like lesions. These arteries ultimately drained into only a short segment patent distal splenic artery located in the splenic hilum (Figure 3). The patient's portal vein was patent, and there was no evidence of cirrhosis.

Throughout his hospital stay, the patient had multiple blood transfusions, and surgery was consulted to do a splenectomy for definitive management of gastric varices and left-sided portal hypertension. His hemoglobin stabilized at 7.9 g/dl, and he was taken to the operating room. Intra-operatively, the spleen was resected and multiple gastric and portosystemic varices originating from the spleen were ligated. During the surgery, the patient started to have massive hematemesis. An EGD was done urgently in the operating room, which showed that there was a large amount of bright red blood present in the distal esophagus. Upon entering the stomach, endoscopic visualization was not possible due to the large volume of bright red blood and clots filling the stomach. The patient was then transferred to interventional radiology for angiogram and embolization.

**Figure 4:**
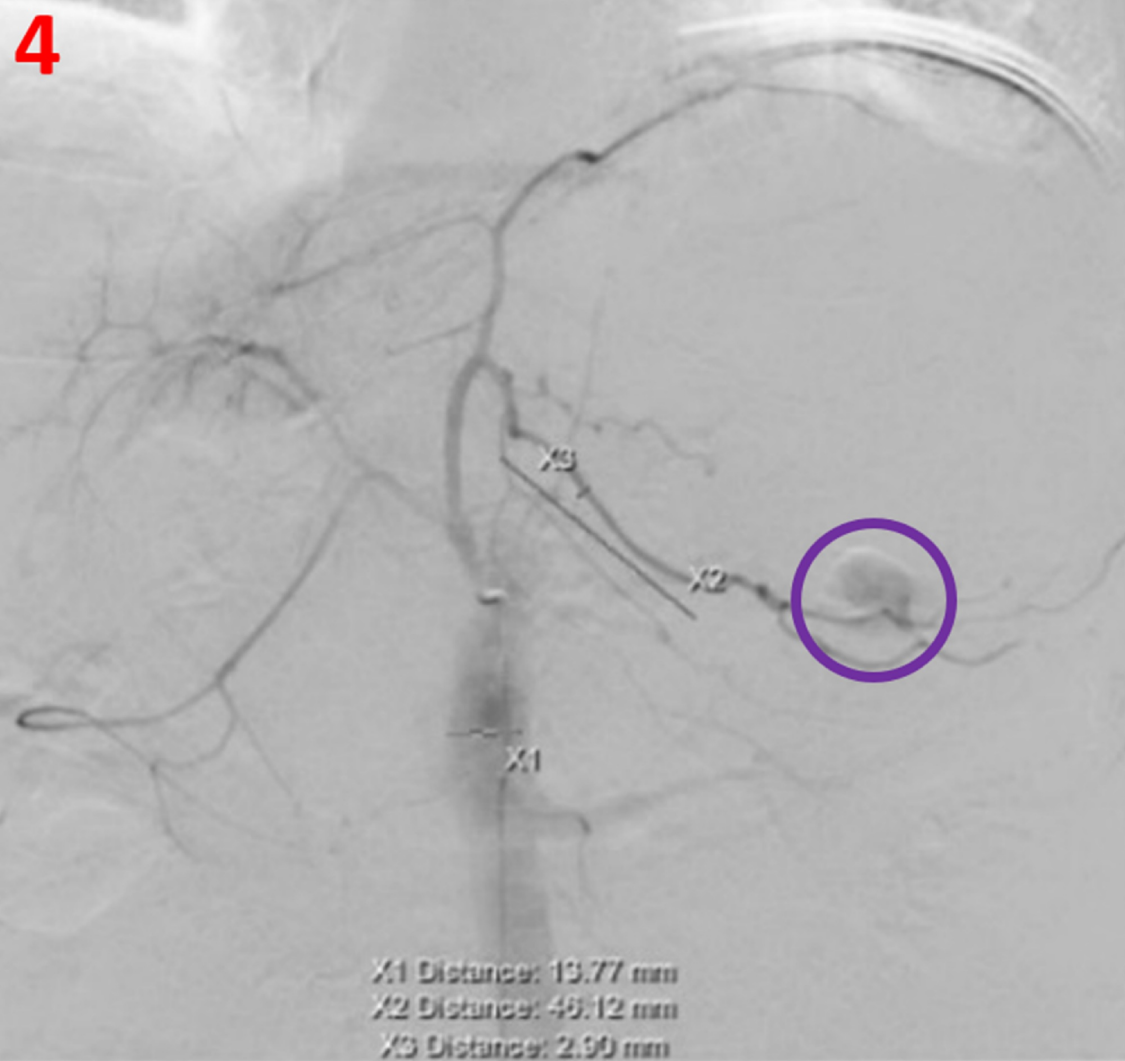
Celiac artery angiogram Celiac artery angiogram shows active extravasation (purple circle) at a branch artery originating from the left gastric artery located along the greater gastric curvature

**Figure 5:**
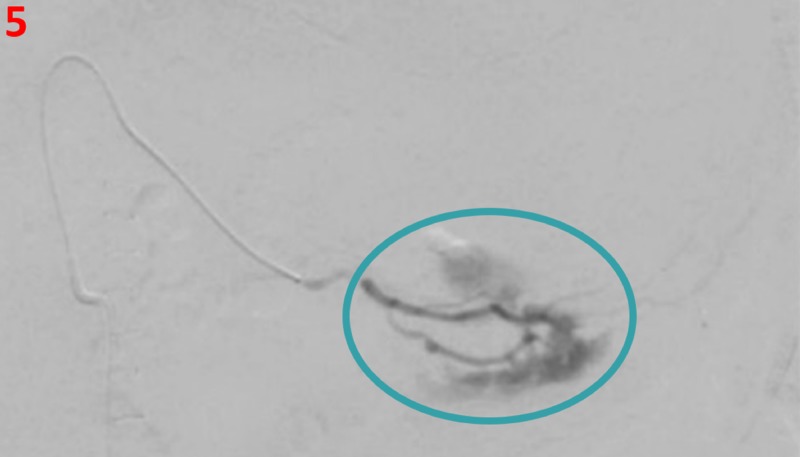
Left gastric artery angiogram Left gastric artery angiogram shows active contrast extravasation (teal circle) consistent with active bleeding from a segmental left gastric artery branch. This vessel is hypertrophied and dilated consistent with Dieulafoy-like lesion

**Figure 6:**
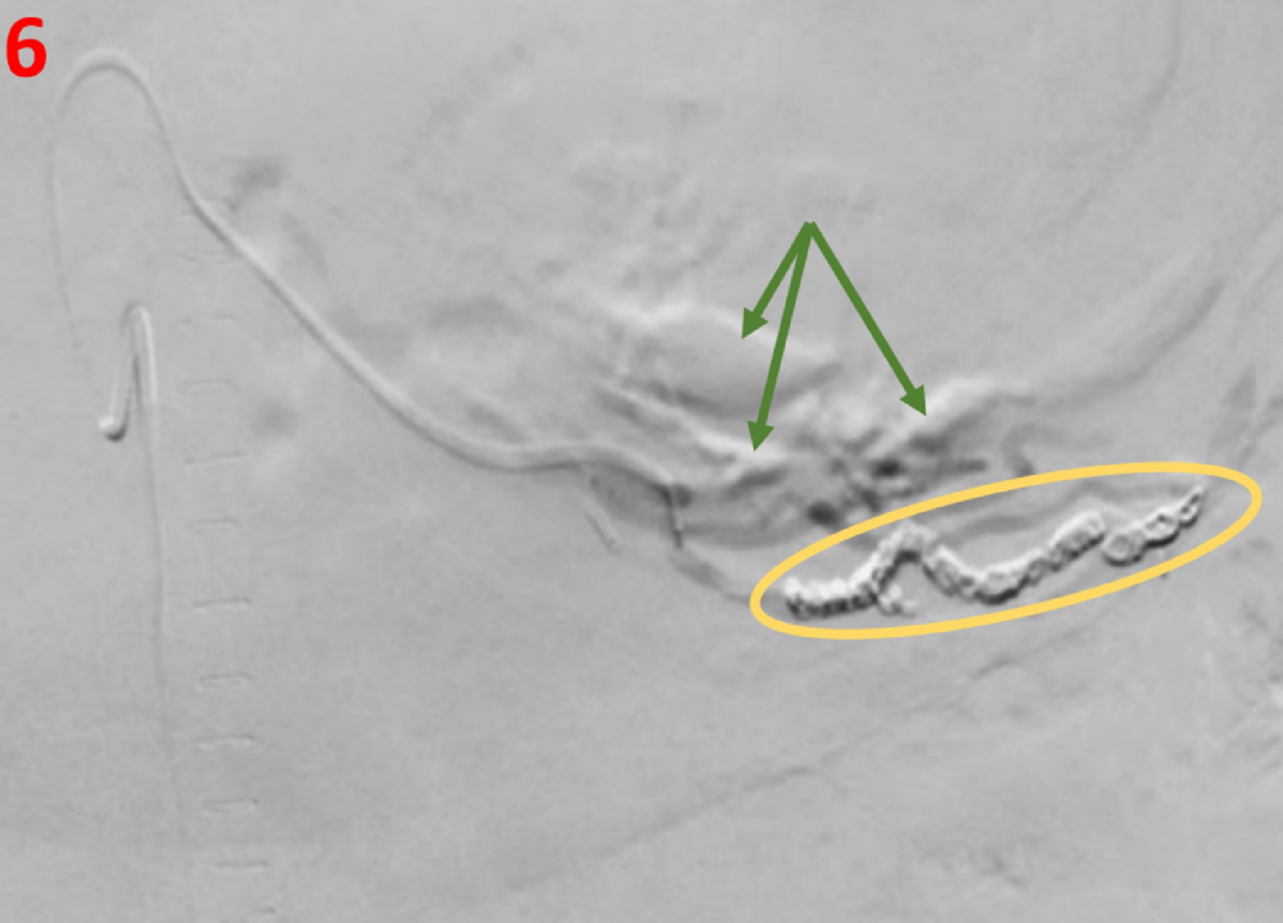
Fluoroscopy image post-intervention Fluoroscopy shows no further extravasation post-Gelfoam and coil embolization (yellow oval). We also see multiple dilated and tortuous arteries (green arrows) arising from the left gastric artery consistent with additional gastric Dieulafoy-like lesions

Initial celiac artery angiogram demonstrated a large focus of active extravasation consistent with active bleeding, originating from a markedly dilated branch artery originating from the left gastric artery located along the greater gastric curvature (Figure 4). Selective angiograms demonstrated feeders from both the left gastric and gastroepiploic arteries (Figure 5). Due to extensive extravasation, the exact source of the bleed was uncertain. Initially, Gelfoam embolization was performed proximal to the active extravasation to eliminate collateral inflow from the gastroepiploic artery and, subsequently, multiple metallic coils were deployed across this branch of the left gastric artery, resulting in the resolution of the extravasation (Figure 6). Additional branches of the left gastric and gastroduodenal artery were also evaluated without evidence of extravasation or pseudoaneurysm. 

After the patient was transferred to the ICU, he developed worsening leukocytosis; on post-op day 2, the patient became septic with concern for gastric ischemia. The patient was taken back to the operating room for an exploratory laparotomy. The surgical team discovered partial necrosis of the stomach wall in the greater curvature and surrounding omentum on their exploratory laparotomy, and afterward, partial gastrectomy and omentectomy were done. After the procedure, the patient developed acute respiratory distress syndrome (ARDS) and acute kidney injury and was initiated on hemodialysis with a continuation of his mechanical ventilation. The following day, the patient continued to be septic and went into multiple organ dysfunction syndrome and was soon pronounced dead, with a standing "do not resuscitate" order by his family.

## Discussion

A unique feature of this case was that the patient had both splenic vein and splenic artery thrombosis, with a viable spleen exclusively supplied by the left gastric artery. This resulted in markedly hypertrophied collateral arteries that traversed through the stomach wall, consistent with Dieulafoy lesions. These hypertrophied arteries ultimately drained into only a short segment patent distal splenic artery located in the splenic hilum. It is probable that by having a splenectomy and ligating the distal splenic artery, the splenic vein, and multiple splenic varices, the pressure was increased in the left gastric arteries, causing them to rupture. This exemplifies a case of complex vasculature and highlights the fact that abrupt changes in hemodynamics do not fare well in critically ill patients. It is well-documented that patients with splenic vein thrombosis associated with active variceal bleeding who do not respond to conservative management need urgent splenectomy to stabilize hemodynamics. Splenectomy normally removes the pressure from the varices by lowering venous outflow of collaterals, but this does not account for concurrent splenic arterial thrombosis, as in our case, where the bleeding was exclusively related to a ruptured artery [5]. In this patient, left gastric artery embolization or partial gastrectomy could have been considered prior to splenectomy to decrease the likelihood of a Dieulafoy-like lesion GI bleed.

Conservative therapy options for left-sided portal hypertension bleeding with low success rates include band ligation, balloon tamponade, vasoconstrictive therapy, and endoscopic treatments including thrombin, glue, or sclerotherapy injections. Cyanoacrylate (glue) has better hemostasis and decreased re-bleeding rates in comparison to other sclerotherapy agents. It is also recommended as first-line therapy, but its use can lead to peripheral embolization [6,7]. It is important that the radiology and surgery communities are aware of the imaging findings and possible vascular complications. To our knowledge, this is the first report that describes this unusual scenario with an unfortunate outcome.

## Conclusions

Concurrent splenic vein and artery thrombosis promoting the formation of a Dieulafoy-like lesion is an extremely rare finding and, to our knowledge, no similar case has been reported in the literature so far. There are very few reports on the management of Dieulafoy and Dieulafoy-like lesions in literature, which contrasts with the availability of a copious amount of data on the management of their impending venous equivalent, variceal bleeding. Factoring in the hemodynamics of Dieulafoy and Dieulafoy-like lesions might deepen the complexity of an intuitive surgical or interventional procedure for an experienced operator, and hence, the appropriate management of this condition requires a collaborative effort between surgical, interventional, and diagnostic services.
